# High-quality microresonators in the longwave infrared based on native germanium

**DOI:** 10.1038/s41467-022-32706-1

**Published:** 2022-10-06

**Authors:** Dingding Ren, Chao Dong, Sadhvikas J. Addamane, David Burghoff

**Affiliations:** 1 Department of Electrical Engineering, University of Notre Dame, Notre Dame, IN USA; 2 Department of Electronic Systems, Norwegian University of Science and Technology (NTNU), Trondheim, Norway; 3 Center for Integrated Nanotechnologies, Sandia National Laboratories, Albuquerque, NM USA

**Keywords:** Mid-infrared photonics, Microresonators

## Abstract

The longwave infrared (LWIR) region of the spectrum spans 8 to 14 μm and enables high-performance sensing and imaging for detection, ranging, and monitoring. Chip-scale LWIR photonics has enormous potential for real-time environmental monitoring, explosive detection, and biomedicine. However, realizing technologies such as precision sensors and broadband frequency combs requires ultra low-loss and low-dispersion components, which have so far remained elusive in this regime. Here, we use native germanium to demonstrate the first high-quality microresonators in the LWIR. These microresonators are coupled to partially-suspended Ge waveguides on a separate glass chip, allowing for the first unambiguous measurements of isolated linewidths. At 8 μm, we measured losses of 0.5 dB/cm and intrinsic quality (*Q*) factors of 2.5 × 10^5^, nearly two orders of magnitude higher than prior LWIR resonators. Our work portends the development of novel sensing and nonlinear photonics in the LWIR regime.

## Abstract (web summary)

Developing longwave infrared technology hide intrinsic challenges but at the same time is important to develop sensing and imaging for detection, ranging, and monitoring systems. Here the authors demonstrate the fabrication of high-quality microresonators in the LWIR with the simple use of native germanium.

## Introduction

Optical microresonators are an essential tool for nonlinear optics. By storing high energy in a small volume, they significantly enhance nonlinear light-matter interactions. A number of interesting effects can be demonstrated in them: frequency comb generation^1,2^, ultra-sensitive sensing^3,4^, optomechanics^5,6^, and non-Hermitian physics^7–9^, to name a few. In the near-infrared, platforms such as SiO_2_ microtoroids^10^ and Si_3_N_4_ ring resonators^11^ can have *Q* factors of billions, while at longer-wavelength midwave infrared, *Q* values can be sustained at the level of several million^12^. Still, there is significant interest in pushing to even longer wavelengths. The rovibrational transition of molecules in the LWIR can be orders of magnitude stronger than shorter wavelengths^13–16^, and of course most blackbody radiation occurs in this range, which is why forward-looking infrared imaging has become so prominent.

While high-power sources like CO_2_ lasers and quantum cascade lasers (QCLs)^17–21^ are readily available at LWIR, chip-scale nonlinear photonics remains challenging. The scaling with the wavelength becomes unfavorable and almost all materials become lossy. Chalcogenides become moderately lossy beyond 7 μm^22^, and silicon has significant absorption loss over 1 dB/cm beyond 7 μm due to multiphonon absorption^23–25^. So far, the lack of high-*Q* microresonators and correspondingly low-loss waveguides suitable for LWIR has remained a bottleneck for LWIR photonics. Despite excellent etch profiles and high-quality epitaxy^26–28^, none have even scanned a laser across a resonance and observed clear features much narrower than the free spectral range, something routine at shorter wavelengths. Germanium is a legendary material platform in which the first transistors were realized, and it is fairly compatible with mainstream silicon technology^29^. With the highest refractive index (*n*_0_ = 4), a large nonlinear refractive index (*n*_2_ = 20 nm^2^/W)^30^, and a wide transparency window (from 2 to 14 μm), Ge shows excellent potential as a low-loss material platform at LWIR^31,32^. However, fabrication of Ge-based on-chip photonic devices also requires compatible cladding materials that confine the light. Recently, epitaxially-grown Ge-on-GaAs and Ge-on-Si has been used to make integrated waveguides and ring resonators^28,33–37^. The optical mode was primarily confined in the Ge part, achieving waveguide losses of 4 dB/cm at LWIR. Specifically, a 4 μm wide Ge-on-Si rib waveguide showed losses of 2.7 dB/cm at 8 μm and 1 dB/cm at 10 μm, while a graded SiGe waveguide showed losses of 3 dB/cm at 8 *μ*m^38,39^. However, these losses are significantly higher than the intrinsic losses of high-quality Ge grown by the traditional Czochralski crystal pulling method under thermodynamic equilibrium conditions. In fact, the losses of pure germanium are so low (<0.15 cm^−1^) that high-quality measurements of the losses were only performed recently^40^. Although epitaxial Ge can be atomically smooth, whenever GaAs or Si are used as the substrate for heteroepitaxy, inevitable degradation of the LWIR transparency results. For Ge grown on GaAs, the volatile arsenic background and the Ga interdiffusion causes unwanted impurity incorporation, lowering the optical transparency at the LWIR^41^. Although Ge grown on Si will not be impacted by the unwanted background impurity incorporation as in GaAs, there is a significant lattice mismatch between Si and Ge. This results in a high density of threading dislocations and significantly lower material quality^42^. The thick waveguides needed for the LWIR excacerbate this issue, and the high index of silicon allows light to penetrate into silicon.

In this work, we utilize the highest-quality, lowest-loss Ge material in conjunction with mechanical fabrication processes to fabricate extremely high-*Q* whispering gallery mode (WGM) resonators and low-loss waveguides. The structures are initially bonded to anodic glass substrates (essentially creating thick Ge-on-insulator), but the glass adjacent to Ge is mostly removed, resulting in structures that are ultimately overhung. These microresonators have ultra-smooth surfaces and are easily separated from their substrates using conventional etchants. To evaluate the optical properties of the resonator, we perform transmission measurements by focusing the emission of an 8 μm distributed feedback (DFB) QCL into a semi-suspended Ge waveguide, which is coupled into the microresonator. By sweeping the wavelength of the QCL, a loaded *Q*_*l*_ of 2.2 × 10^5^ and an intrinsic *Q*_*i*_ of 2.5 × 10^5^ are measured. This is similar for both the transverse electric (TE) and transverse magnetic (TM) polarizations.

## Results

### Fabrication process and devices

**Fig. 1:**
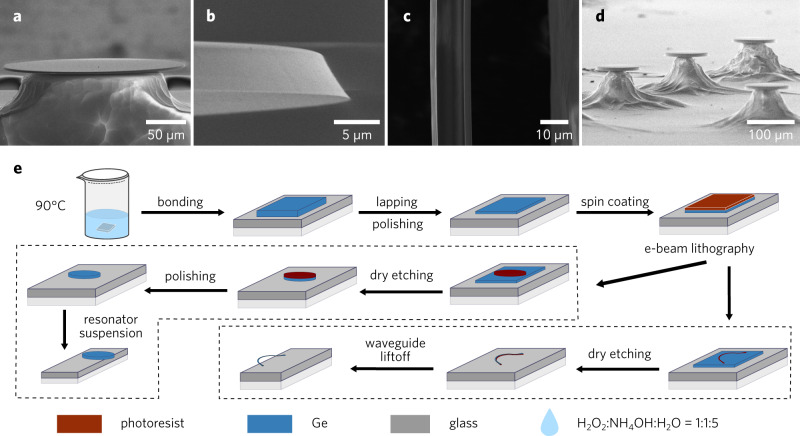
Images and fabrication of the non-epitaxial Ge microresonators and suspended waveguides. **a** SEM image of a non-epitaxial Ge microresonator. The 450 μm disk is supported by a glass pillar. **b** Magnified view of the microresonator edge where the WGM mode resides. **c** Top view of the suspended part of the Ge waveguide (Supplementary Fig. S3). The waveguide is on the left and the glass substrate is on the right. **d** SEM images of a WGM microresonator array. **e** Overview of the fabrication procedure, which relies on a combination of wafer bonding and polishing processes.

Figure 1a–d shows scanning electron microscope (SEM) images of the fabricated devices. A microresonator with diameter of 450 μm can be seen in Fig. 1a. The resonator has a uniform thickness across the whole resonator, while the outermost 100 μm is suspended away from the dune-shaped glass supporting structure in the center. This eliminates the losses due to the glass substrate at LWIR. To look at the active part of the WGM microresonator, we zoomed in to the edge of the WGM microresonator. Figure 1b shows a zoomed image of the edge. It is angled inward by about 30-degrees, a consequence of isotropic etching and edge rounding from the polishing process. By changing the plasma strength with controlled anisotropic etching and tuning the polishing pressure, the geometry could be further tuned, with potential for dispersion engineering^43^. To efficiently couple LWIR light into the WGM microresonator, we fabricated a partially-suspended Ge waveguide on a glass chip with dimensions of 4 × 2 mm^2^, shown in Fig. 1c. The waveguide is around 5 μm thick, similar to that of the microresonator, and is 10 μm wide. The curved part of the suspended waveguide shows uniform geometry and smooth surface, making it ideal for coupling measurements.

Figure 1e shows an overview of the fabrication process of the microresonators and of the partially-suspended waveguides. The details are summarized in the Methods, but briefly it relies on a combination of etching and post-polishing. The bottom side of the WGM microresonator has only gone through the wafer bonding process and has the same surface roughness as the original polished Ge wafer, and we did not find a significant difference in the surface roughness between the side-facet and the bottom facet. We performed atomic force microscopy in tapping mode in the center of the WGM microresonator to evaluate the surface roughness of polished Ge surface. Only sparsely distributed spot-like extrusions with the height of tens of nanometers could be found over the 13.5 × 13.5 μm^2^ area (Supplementary Fig. S2). The surface roughness of polished WGM microresonator is about 2 orders of magnitude smaller than the wavelength in LWIR, so we can safely ensure that there is no significant surface roughness-induced scattering loss for our WGM microresonator at the LWIR. In addition, we show in Fig. 1d a fabricated Ge microresonator array, proving the versatility and robustness of the non-epitaxial Ge platform.

### Optical simulation

**Fig. 2:**
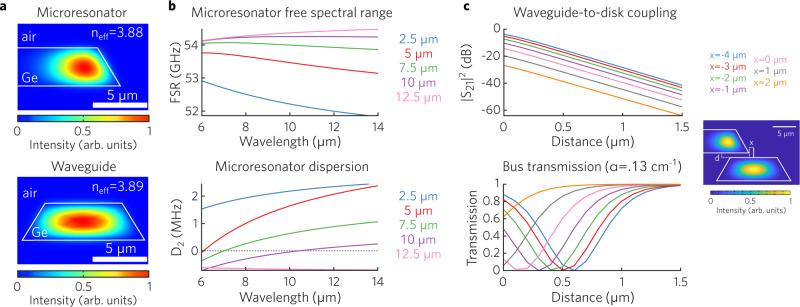
Simulations of mode, dispersion and coupling process. **a** FEM simulation of the guided quasi-TE mode in the Ge WGM microresonator from Fig. 1a and Ge waveguide from Fig. 1c. **b** Calculated free spectral range and dispersion versus waveguide thickness for a 450 μm diameter waveguide. Low anomalous dispersion (positive *D*_2_) is only possible with very thick waveguides, in the 7.5–10 μm range at wavelength of 8 μm. **c** Simulated coupling efficiency and bus transmission versus vertical distance between the microresonator and waveguide from a. Different curves indicate different radial positions (x) of the WGM microresonator and waveguide.

To simulate the light propagation in the microresonator, the waveguides and their coupling behavior, we performed finite element method (FEM) simulations based on the geometry and dimensions shown in Fig. 1. The effective refractive indices of 3.88 and 3.89 were calculated for the fundamental quasi-TE mode in the WGM microresonator and Ge waveguide, respectively, with each mode profile shown in Fig. 2a. To fully explore the potential of LWIR Ge photonics at 8 μm, we calculated the free spectral range (FSR, a.k.a. *D*_1_) and the change in FSR per mode (*D*_2_), which are commonly used to characterize microcavities for nonlinear optics. They are defined by

1 $${\omega }_{\mu }={\omega }_{0}+{D}_{1}\mu+\frac{1}{2}{D}_{2}{\mu }^{2}+\cdot \cdot \cdot$$

where *μ* is the mode number and *ω*_*μ*_ is the *μ*th frequency^44^. The calculations were performed using the simulations of WGM microresonators with thicknesses ranging from 2.5 μm, the typical thickness of epitaxial Ge-on-Si^38^, up to 12.5 μm. As is shown in Fig. 2b, there is a significant increase of the FSR with increasing thickness of the WGM microresonator (over the entire spectrum LWIR range). Similarly, with increasing thickness *D*_2_ goes from positive to negative. This tuning range is several orders of magnitude higher than that of the SiO_2_ WGM microresonators, a result of the significant refractive index contrast between Ge and air^43^. Note that achieving low and flat anomalous dispersion with these resonators requires Ge thicknesses in the 7.5-10 μm range, which is very difficult using epitaxially-grown samples on non-native substrates. To further explore the coupling, we used coupled mode theory^45^ together with the simulated mode profiles in Fig. 2a to calculate the coupling between waveguide and disk (top of Fig. 2c) as a function of waveguide position. As the waveguide is moved closer to the resonator mode, the coupling increases drastically. We also calculate the transmission through the waveguide when it is coupled to a cavity with intrinsic modal losses of .13 cm^−1^ (bottom of Fig. 2c). When the waveguide is close to the disk mode, the external coupling losses match the internal losses and critical coupling is achieved, causing the transmission to become zero.

### Optical setup and measurements

**Fig. 3:**
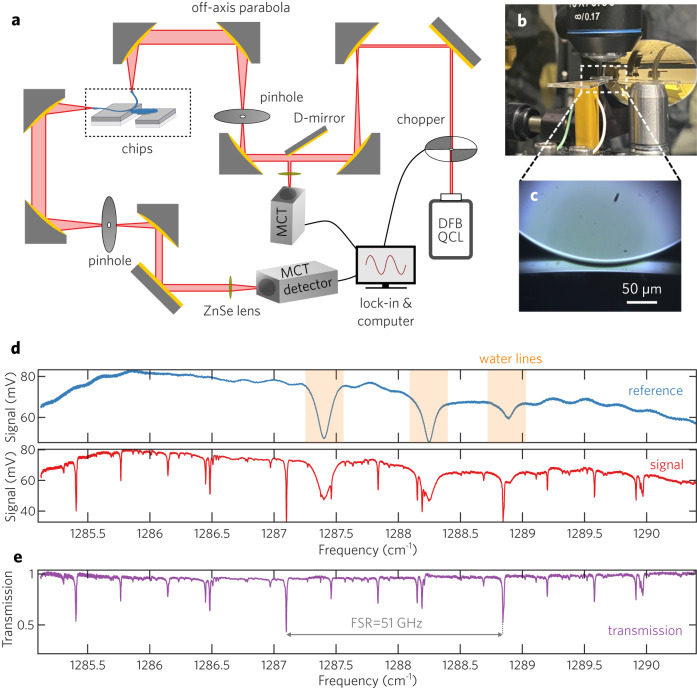
Experimental setup for transmission measurements. **a** Schematic of the optical path and components. A DFB QCL is focused into the waveguide and coupled to the resonator. **b** Photograph of the coupling setup. The glass chip with microresonator is mounted on the piezoelectric actuator in yellow and waveguide chip is mounted on the metallic post on the right. **c** Optical image of the Ge WGM and waveguide when coupled, taken from the objective above the coupling area shown in 3b. **d** Two consecutive wavelength-tuning scans when the microresonator from Fig. 1a was moved away from the waveguide (reference) and approached the waveguide. **e** Normalized transmission scan from Fig. 3d.

To characterize the microresonators, we performed direct transmission measurements using a single-mode DFB QCL. The wavelength of the QCL can be tuned from 1285.5 to 1290.5 cm^−1^ by controlling both the QCL current and temperature. As is shown in the schematic of the optical path and components in Fig. 3a, the collimated laser beam was expanded through two off-axis parabola (OAP) pairs by a factor of ten and two times, consecutively, reaching a beam size of about 30 mm. Next, the collimated beam was focused on the fabricated Ge waveguide entrance facet using 1 inch OAP mirror, photographed in Fig. 3b, reaching a beam size of about 20 × 20 μm^2^. The transmitted laser signal from the waveguide was collected by an OAP of 4-inch focal length, and the collimated beam size was reduced by two times using a pair of OAPs. The transmitted signal was collected using a Mercury-Cadmium-Telluride (MCT) detector connected to a lock-in amplifier system to eliminate background. We further used two pinholes at the confocal planes of the OAP mirror pairs to exclude unwanted interference from the scattered laser signal in the system. These were aligned visually using an electronic autocollimator. In conjunction with the fact that the waveguide is bent by 90 degrees (see Supplementary Fig. S3 for a more detailed picture), no scattered light was observed. The waveguide chip was mounted on a piezoelectric actuator that allowed fine adjustment of the distance between the waveguide and the resonator.

Figure 3d shows a pair of representative scans of waveguide with and without the coupling to the WGM microresonator. In the reference scan, the three dips of the transmission signals match well to the H_2_O gas lines at 1287.40 cm^−1^, 1288.25 cm^−1^, and 1288.89 cm^−1^ (Supplementary Fig. S4). We use these three gas absorption features as fine calibration for the QCL wavelength. When the WGM microresonator is coupled to the waveguide, we observed a series of sharp dips much narrower than the three H_2_O gas absorption lines. These come from coupling between the waveguide and the WGM microresonator. To acquire the transmission information without interference from the signal variation of the background, we divide the signal by the reference; the normalized transmission is plotted in Fig. 3e. The most prominent dip corresponds to coupling to the fundamental whispering gallery mode, repeating three times when the laser is scanned from 1285 cm^−1^ to 1290 cm^−1^. This indicates a free spectral range of 51 GHz, which matches well with the calculation based on the FEM simulation. Smaller dips are present as well (with similar FSR), indicating coupling to higher-order mode families. Coupling to these modes can be strengthened or suppressed by adjustment of the waveguide’s radial position. From the FSR, we estimate an effective group index of 4.15.

**Fig. 4:**
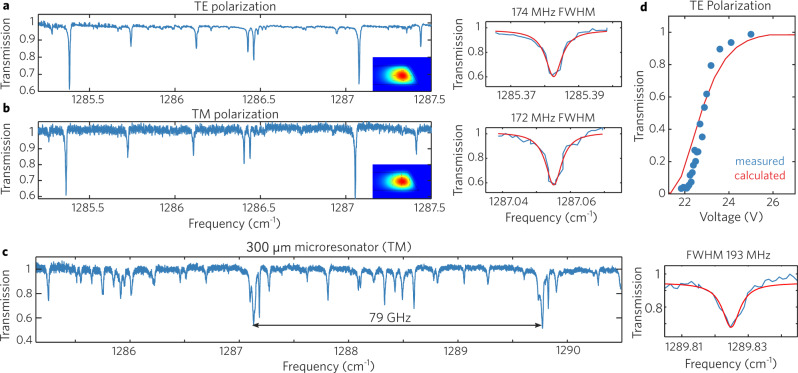
Polarization-dependent coupling measurements. **a** TE polarization transmission of microresonator from Fig. 1a, with a FWHM linewidth of 174 MHz. **b** TM polarization transmission of microresonator from Fig. 1a, with a FWHM linewidth of 172 MHz. **c** Transmission measurement of a second WGM microresonator with diameter of 300 μm using laser of TM polarization, with a FWHM linewidth of 193 MHz. **d** Trend of transmission (TE polarization) versus the piezoelectric actuator voltage. The WGM microresonator was mounted on the piezoelectric actuator in Fig. 3b, and the vertical distance between the microresonator and the waveguide can be tuned by changing the applied voltage of the piezoelectric actuator. Due to the very high index mismatch between germanium and air, critical coupling is achieved only over a very short range.

To evaluate the properties of the WGM microresonator, the orientation of the QCL was adjusted to generate either TE- or TM-polarized laser emission, and this was fed into the optical path for the transmission measurement. Figure 4a, b show the result of both the TE and TM transmission measurements, respectively, and at this distance each show two central transmission dips of about 60% separated by an FSR. A Lorentzian model was used to fit the dips at 1285.4 cm^−1^ and 1287.1 cm^−1^, giving a full width half maximum (FWHM) values of 174 MHz and 172 MHz, respectively. This corresponds to a loaded *Q*_*l*_ of 221,000 and 224,000 for the respective TE and TM polarizations. Figure 4c shows the transmission scan of a 300 *μ*m diameter resonator, which leads to an FSR of 80 GHz (in theory) and 79 GHz (in practice). This proves that the dips come from the coupling between the microresonator and the waveguide.

To investigate the coupling condition, we performed a series of transmission measurements by changing the voltage of the piezoelectric actuator, which in turn changes the separation between the WGM microresonator and the waveguide. Figure 4d shows the transmission versus piezoelectric actuator voltage for TE polarization, and we can see a monotonic decrease of the transmission by reducing the voltage of the piezoelectric actuator. If we convert the voltage change to the distance of movement by estimating travel per distance ratio of 0.2 *μ*m/V of the piezoelectric actuator, we can compare this to the simulations in Fig. 2. From this plot, we can infer that the 60% transmission dips are in an under-coupled condition. Those additional transmission dips with smaller coupling strengths correspond to coupling with higher-order modes, which start to grow significantly as the waveguide is brought closer (Supplementary Fig. S5), suggesting parasitic coupling between the waveguide and higher-order microresonator modes^46^. These mode interactions can induce anomalous GVD in normal dispersion devices and lower the threshold for Kerr frequency comb generation^47–50^. Further development of a narrower single-mode coupler will enable coupling at a greater microresonator-waveguide spacing. The intrinsic *Q* factor can be calculated using^25^

2 $${Q}_{i}=\frac{2{Q}_{l}}{1+\sqrt{T}}$$

where *Q*_*l*_ is the loaded *Q* factor and T is the transmission when undercoupled. Using this, we calculate intrinsic *Q* factors of 251,000 and 253,000 for the TE and TM polarization, respectively. There is little difference between these *Q*s, suggesting that all surfaces of the WGM microresonators are equally smooth. We fitted 12 of the transmission dips at 1285.38 and at 1287.06 cm^−1^ in under-coupled condition, and the averaged *Q* factor is 2.2 × 10^5^ (Supplementary Fig. S6). These high-*Q* values are almost two orders of magnitude higher than those from previous microresonators in the LWIR, which are summarized in Table 1.

**Table 1:** Comparison of previous LWIR microresonators

Material	Synthesis	Type	*Q* factor	Radius	Wavelength	Modal area	Coupling extinction ratio	Device concept
Diamond^27^	CVD	WGM	3,648	500 μm	9.6 μm	N.A.	N.A.	Fiber and resonator chip
SiGe^26^	LEPECVD	Ring resonator	3,200	100 μm	8 μm	~20 μm^2^	max 10 dB	One chip
Ge^28^	MBE	Ring resonator	2,000 to 10,000	500 μm	8-11 μm	~10 μm^2^	max 10 to 15 dB	One chip
This work	Czochralski	WGM	250,000	225 μm	7.8 μm	20.1 μm^2^	max 15 dB	Two chips with tunable coupling

## Discussion

From these intrinsic *Q*s, we can now directly estimate the effective loss of the mode using $\alpha=\frac{2\pi {n}_{g}}{Q{\lambda }_{0}}$, giving an intrinsic loss of 0.13 cm^−1^ (0.5 dB/cm). This value is very close to the reported losses of germanium in this range, showing that these resonators have the highest quality factors that are possible for this material (Supplementary Fig. S7). The loss of the suspended portion of the waveguides is likely similar, although the larger losses of the glass-clad portion makes the averaged measured losses to be 9 cm^−1^ (Supplementary Fig. S3). At even longer wavelengths, beyond the onset of Ge’s three-phonon absorption, it may be beneficial to further explore alternative materials such as diamond and KBr using suspended structures. Still, Ge is quite competitive in these wavelengths due to its combined low loss and fabrication feasibility. Integration of Ge-on-diamond could also be an appealing combination for monolithic low-loss Ge waveguides.

With this development, many of the microresonator-based technologies that were previously not viable in the LWIR are now feasible. For example, an attractive application for this platform is for chip-scale ultrabroadband sources pumped by QCLs, like Kerr comb generation (in the resonator geometry). While combs can be directly generated by QCLs^17,19–21^, achieving spectral bandwidths over 100 cm^−1^ is very challenging. The extremely wide transparency range of germanium makes it attractive for this application, as with it octave-spanning operation could be feasible. While in this work we did not attempt Kerr frequency comb generation as the inefficient coupling into and out of the waveguide precluded this, it is worth calculating the parametric threshold for frequency comb generation. Assuming a modal diameter of 5 μm, we calculate that the threshold for comb generation should be approximately 240 mW under optimal conditions^51^. This threshold is well below the 10 W range produced by state-of-the-art LWIR QCLs^52^. Still, achieving fully-integrated comb generation in the current implementation of this platform would be difficult due to the suspension required even for the waveguide. Though the waveguides are fairly lossy when on the glass, they are actually remarkably low when one considers that the losses of glass can exceed 10,000 cm^−1^ in the LWIR. This is a result of the large index mismatch of germanium and the glass, leading to a small overlap of the mode with the lossy region. Predeposition of a thin film of a moderately low-loss low-index material, such as ZnS, would drastically reduce the loss to a similar level as the disks (Supplementary Fig. S3f).

In conclusion, we presented a non-epitaxial fabrication platform for germanium microresonators and waveguides whose quality factors are two orders of magnitude higher than prior work. This methodology avoids the defects inevitably associated with thick epitaxial growth. Using a single-mode DFB QCL, we observed transmission dips with repetition rate matched well to the FSRs of the WGM microresonators. Loaded *Q* factors of 221,000 and 224,000 were measured for the TE and TM polarization, respectively, corresponding to the intrinsic *Q* factors of 2.5 × 10^5^. These excellent *Q* values represent a milestone for the low-loss LWIR photonics, and we expect similar platforms to be essential for future chip-scale sensing and nonlinear photonics.

## Methods

### Wafer bonding process

A sample of Hoya SD-2 anodic bonding glass of 1.5 × 1.5 cm^2^ in size and 1 mm in thickness was firstly cleaned by acetone and isopropanol. This was followed by a hot bath in H_2_O_2_:NH_4_OH:H_2_O (volume ratio of 1:1:5) at 90 ^∘^C for 15 min. This step ensures that the surface of the glass has high surface energy, favorable for hydrophilic bonding^53^. A piece of highly resistive Ge of 1 × 1 cm^2^ in size and 500 μm in thickness was dipped into the same H_2_O_2_:NH_4_OH:H_2_O solution (at room temperature for 5 s). Any surface treatment of Ge at a higher temperature or for a longer time will lead to surface degradation. After the surface treatment, the glass and Ge piece were sandwiched into graphite plates inside a thermocompression bonder. 3000 N force was applied, and the bonder was heated to 300 ^∘^C for 60 min (Supplementary Fig. S1).

### Nanofabrication process

After the mechanical lapping and polishing process, which utilized adhesive lapping to minimize scratch formation, electron beam lithography (EBL) was used to define the features of the Ge, using a Vistec EBPG5200 EBL System at 100 KV. HMDS adhesion promoter and Espacer-300Z discharger were applied before and after the spin-coating of the e-beam resist MA-N-2403. This step is essential. An SF_6_ chemistry in a deep RIE system was used to etch through the Ge layer to define the features of the microresonator. To ensure all the facets of the resonator were finished with a smooth surface, the etched microresonator was polished after the nanofabrication. The sample was then iteratively etched by alternatively HF acid etching and a sidewall passivation process; this allows us to etch back the glass layer from the edge of the resonator. This was performed three times with 49% HF for 1 min. By using the microresonator itself as the photolithography mask, a flood exposure with positive S1813 photoresist was performed to passivate the vertical sidewall of the microresonator and its supporting structure. This iteration of etching and passivation prevented severe in-plane etching that could easily wash away the microresonator and maintained effective etching speed in the vertical direction.

The curved Ge waveguides were fabricated using the same process in order to efficiently couple the light from free space into the microresonator. The Ge waveguide has a larger in-plane end facet to enhance the collection of the light from the free space. Both the coupling sections of the Ge microresonator and the waveguide were diced to the edge of the glass chips (Supplementary Fig. S8 and Section VI). A final HF etching process was used to further etch back the glass, suspending both the Ge microresonator and the Ge waveguide from the glass chips.

### Tuning of the quantum cascade laser

A DFB QCL with peak power of 250 mW and wavelength tuning range from 1285.5 to 1290.5 cm^−1^ was used to perform the wavelength-dependent transmission measurements. A linear parameter space of current and temperature was established in order to wavelength-tune at a constant power of 10 mW. An Arroyo 6310-QCL controller was used to control the temperature of the TEC and current of the QCL laser, with a minimum step size of 0.01 ^∘^C and 0.02 mA.

## Supplementary information

Supplementary

## Data Availability

The data and code that support the findings of this study are available in Figshare with the identifier 10.6084/m9.figshare.20369253.

## Code availability

The data and code that support the findings of this study are available in Figshare with the identifier 10.6084/m9.figshare.20369253.

## References

[1] Del’Haye P (2007). Optical frequency comb generation from a monolithic microresonator. Nature.

[2] Gaeta AL, Lipson M, Kippenberg TJ (2019). Photonic-chip-based frequency combs. Nat. Photonics.

[3] Armani AM, Kulkarni RP, Fraser SE, Flagan RC, Vahala KJ (2007). Label-free, single-molecule detection with optical microcavities. Science.

[4] Guggenheim JA (2017). Ultrasensitive plano-concave optical microresonators for ultrasound sensing. Nat. Photonics.

[5] Anetsberger G, Rivière R, Schliesser A, Arcizet O, Kippenberg TJ (2008). Ultralow-dissipation optomechanical resonators on a chip. Nat. Photonics.

[6] Wiederhecker GS, Chen L, Gondarenko A, Lipson M (2009). Controlling photonic structures using optical forces. Nature.

[7] Peng B (2014). Parity–time-symmetric whispering-gallery microcavities. Nat. Phys..

[8] Chang L (2014). Parity–time symmetry and variable optical isolation in active–passive-coupled microresonators. Nat. Photonics.

[9] Cao H, Wiersig J (2015). Dielectric microcavities: Model systems for wave chaos and non-Hermitian physics. Rev. Mod. Phys..

[10] Armani DK, Kippenberg TJ, Spillane SM, Vahala KJ (2003). Ultra-high-Q toroid microcavity on a chip. Nature.

[11] Pfeiffer MH (2018). Ultra-smooth silicon nitride waveguides based on the Damascene reflow process: Fabrication and loss origins. Optica.

[12] Luke K, Okawachi Y, Lamont MR, Gaeta AL, Lipson M (2015). Broadband mid-infrared frequency comb generation in a Si_3_N_4_ microresonator. Opt. Lett..

[13] Brown SS (2003). Absorption spectroscopy in high-finesse cavities for atmospheric studies. Chem. Rev..

[14] Webber ME, Pushkarsky M, Patel CKN (2005). Optical detection of chemical warfare agents and toxic industrial chemicals: Simulation. J. Appl. Phys..

[15] Kasahara R, Kino S, Soyama S, Matsuura Y (2018). Noninvasive glucose monitoring using mid-infrared absorption spectroscopy based on a few wavenumbers. Biomed. Opt. Express.

[16] Jernelv IL (2019). A review of optical methods for continuous glucose monitoring. Appl. Spectrosc. Rev..

[17] Hugi A, Villares G, Blaser S, Liu HC, Faist J (2012). Mid-infrared frequency comb based on a quantum cascade laser. Nature.

[18] Meng B, Wang QJ (2015). Broadly tunable single-mode mid-infrared quantum cascade lasers. J. Opt..

[19] Lu QY (2015). High power frequency comb based on mid-infrared quantum cascade laser at *λ* ~ 9 μm. Appl. Phys. Lett..

[20] Hillbrand J, Andrews AM, Detz H, Strasser G, Schwarz B (2019). Coherent injection locking of quantum cascade laser frequency combs. Nat. Photonics.

[21] Meng B (2020). Mid-infrared frequency comb from a ring quantum cascade laser. Optica.

[22] Feng X (2020). Few-moded ultralarge mode area chalcogenide photonic crystal fiber for mid-infrared high power applications. Opt. Express.

[23] Johnson F (1959). Lattice absorption bands in silicon. Proc. Phys. Soc..

[24] Bendow B, Lipson HG, Yukon SP (1977). Multiphonon absorption in highly transparent semiconducting crystals. Phys. Rev. B.

[25] Miller SA (2017). Low-loss silicon platform for broadband mid-infrared photonics. Optica.

[26] Ramirez JM (2019). Broadband integrated racetrack ring resonators for long-wave infrared photonics. Opt. Lett..

[27] Lee Y-J, Das A, Talghader JJ (2020). High-Q diamond microresonators in the long-wave infrared. Opt. Express.

[28] Kozak DA, Tyndall NF, Pruessner MW, Rabinovich WS, Stievater TH (2021). Germanium-on-silicon waveguides for long-wave integrated photonics: Ring resonance and thermo-optics. Opt. Express.

[29] Carroll L (2012). Direct-Gap Gain and Optical Absorption in Germanium Correlated to the Density of Photoexcited Carriers, Doping, and Strain. Phys. Rev. Lett..

[30] Zhang, L., Agarwal, A. M., Kimerling, L. C. & Michel, J. Nonlinear Group IV photonics based on silicon and germanium: From near-infrared to mid-infrared. *Nanophotonics* **3**, 247–268 (2014).

[31] Hon NK, Soref R, Jalali B (2011). The third-order nonlinear optical coefficients of Si, Ge, and Si_1-x_Ge_x_ in the midwave and longwave infrared. J. Appl. Phys..

[32] Soref R (2010). Mid-infrared photonics in silicon and germanium. Nat. Photonics.

[33] Chang Y-C, Paeder V, Hvozdara L, Hartmann J-M, Herzig HP (2012). Low-loss germanium strip waveguides on silicon for the mid-infrared. Opt. Lett..

[34] Brun M (2014). Low loss SiGe graded index waveguides for mid-IR applications. Opt. Express.

[35] Nedeljkovic M (2017). Germanium-on-silicon waveguides operating at mid-infrared wavelengths up to 8.5 *μ*m. Opt. Express.

[36] Liao, H.-Y. et al. Low-Loss Ge-on-GaAs Platform for Mid-Infrared Photonics. *Conference on Lasers and Electro-Optics*, SM2K.1 (2017).

[37] Montesinos-Ballester M (2020). Ge-rich graded SiGe waveguides and interferometers from 5 to 11 *μ*m wavelength range. Opt. Express.

[38] Gallacher K (2018). Low loss Ge-on-Si waveguides operating in the 8–14 *μ*m atmospheric transmission window. Opt. Express.

[39] Ramirez J (2018). Graded sige waveguides with broadband low-loss propagation in the mid infrared. Opt. Express.

[40] Lee Y-J, Das A, Mah ML, Talghader JJ (2020). Long-wave infrared absorption measurement of undoped germanium using photothermal common-path interferometry. Appl. Opt..

[41] Bai Y, Bulsara MT, Fitzgerald EA (2012). Photoluminescence and secondary ion mass spectrometry investigation of unintentional doping in epitaxial germanium thin films grown on III-V compound by metal-organic chemical vapor deposition. J. Appl. Phys..

[42] Eaglesham D, Cerullo M (1991). Low-temperature growth of Ge on Si (100). Appl. Phys. Lett..

[43] Yang KY (2016). Broadband dispersion-engineered microresonator on a chip. Nat. Photonics.

[44] Herr T (2012). Universal formation dynamics and noise of kerr-frequency combs in microresonators. Nat. Photonics.

[45] Haus H, Huang W, Kawakami S, Whitaker N (1987). Coupled-mode theory of optical waveguides. J. Lightwave Technol..

[46] Del’Haye P, Arcizet O, Gorodetsky ML, Holzwarth R, Kippenberg TJ (2009). Frequency comb assisted diode laser spectroscopy for measurement of microcavity dispersion. Nat. Photonics.

[47] Savchenkov A (2012). Kerr frequency comb generation in overmoded resonators. Opt. Express.

[48] Liu Y (2014). Investigation of mode coupling in normal-dispersion silicon nitride microresonators for kerr frequency comb generation. Optica.

[49] Xue X (2015). Normal-dispersion microcombs enabled by controllable mode interactions. Laser Photonics Rev..

[50] Jang JK (2016). Dynamics of mode-coupling-induced microresonator frequency combs in normal dispersion. Opt. Express.

[51] Xuan Y (2016). High-Q silicon nitride microresonators exhibiting low-power frequency comb initiation. Optica.

[52] Zhou W, Lu Q-Y, Wu D-H, Slivken S, Razeghi M (2019). High-power, continuous-wave, phase-locked quantum cascade laser arrays emitting at 8 μm. Opt. Express.

[53] Dziuban, J. A. *Bonding in Microsystem Technology*, 24 (Springer Science & Business Media, 2007).

