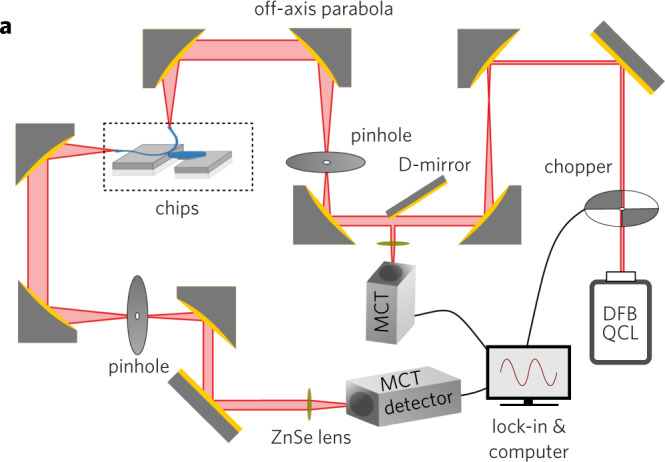# Publisher Correction: High-quality microresonators in the longwave infrared based on native germanium

**DOI:** 10.1038/s41467-022-35137-0

**Published:** 2022-12-19

**Authors:** Dingding Ren, Chao Dong, Sadhvikas J. Addamane, David Burghoff

**Affiliations:** 1 Department of Electrical Engineering, University of Notre Dame, Notre Dame, IN USA; 2 Department of Electronic Systems, Norwegian University of Science and Technology (NTNU), Trondheim, Norway; 3 Center for Integrated Nanotechnologies, Sandia National Laboratories, Albuquerque, NM USA

**Keywords:** Mid-infrared photonics, Microresonators

Correction to: *Nature Communications* 10.1038/s41467-022-32706-1, published online 06 October 2022

Fig 3a in the PDF version of this article inadvertently displayed the off-axis parabolas incorrectly; the figure 3a should have appeared as shown below. The original article PDF has been corrected. The HTML version was unaffected.