# Supplementary material for: High-quality microresonators in the longwave infrared based on native germanium
Source: Nat Commun. 2022 Oct 6;13:5727. doi: 10.1038/s41467-022-32706-1 (PMC9537179; doi:10.1038/s41467-022-32706-1)
Supplement: Supplementary file 1 — Supplementary [file 41467_2022_32706_MOESM1_ESM.pdf]

Supplementary Information

High-quality microresonators in the longwave infrared based on native germanium

Dingding Ren[1,2,*], Chao Dong[1], Sadhvikas J. Addamane[3], and David Burghoff[1]

[1]Department of Electrical Engineering, University of Notre Dame, Notre Dame IN, USA
[2]Department of Electronic Systems, Norwegian University of Science and Technology (NTNU), Trondheim, Norway
[3]Center for Integrated Nanotechnologies, Sandia National Laboratories, Albuquerque NM, USA
[*]e-mail: dren@nd.edu

Contents

I FABRICATION CHARACTERIZATION 2
II WAVEGUIDE DETAILS 3
III WATER VAPOR ABSORPTION LINES 3
IV MODIFYING THE WAVEGUIDE-RESONATOR COUPLING 4
V OPTICAL LOSSES OF SI AND GE IN THE LWIR 4
VI PROTECTIVE DICING 5
References 5

I Fabrication characterization

The wafer bonding was performed using a thermocompression bonder under ambient conditions (Fig. S1a). Pyrolytic graphite was used for sandwiching the glass and Ge wafer. As is shown in Fig. S1b, the bonded interface show uniform contrast instead of the periodic interference fringes shown in Fig. S1c. The interference fringes are signatures of an air gap between the Ge and the glass.

The choice of anodic bonding glass as a substrate was made because it is sufficiently expansion-matched to a wide variety of semiconductors and can be selectively etched. For example, in addition to Ge it could also be well-bonded to GaAs-AlGaAs heterostructures (Fig. S1d,e). While III-Vs are not quite as low-loss in the LWIR as Ge, they have the advantage that their Kerr nonlinearity can be engineered using band-structure engineering. Additionally, electro-optic nonlinearities could be introduced, which are not present in centrosymmetric materials.

Atomic force microscopy (AFM) was used to characterize the Ge surface after the mechanical lapping and polishing procedures. The characterization experiment was performed using a Park XE7 system in tapping/non-contact mode. Representative surface morphology is shown in Fig. S2a and S2b for the top view and 3D view, respectively. The characterized surface shows only spike-like extrusion with a height less than 60 nm, which is two orders of magnitude smaller than the wavelength in LWIR, proving that this is an ultra-smooth process suitable for LWIR photonics.

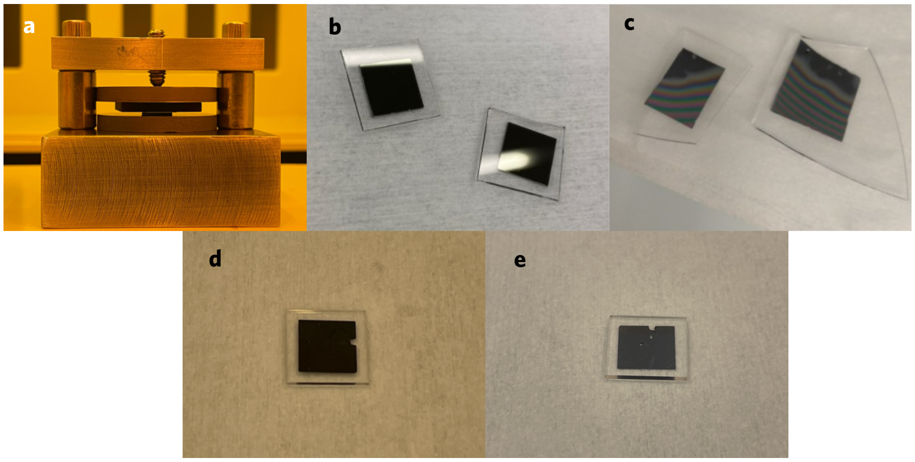

Figure S1. a. Photographic image of the bonder when the Ge and glass have been assembled before the annealing process. **b**. Two fully bonded Ge-on-glass samples. **c**. Two partially-bonded Ge-on-glass samples. **d**, **e**.. Bonding of 10 μm GaAs-AlGaAs heterostructure to anodic glass, viewed from the bottom (d) and top (e).

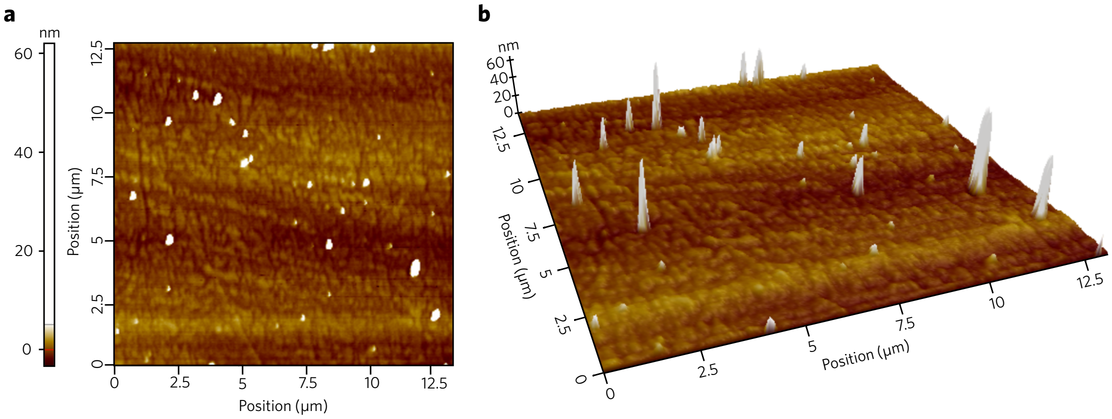

Figure S2. Top-view (a) and 3D-view (b) of atomic force microscopy images of the polished Ge surface. The scanned area is $13.5 \times 13.5\,\mu\text{m}^2$.

II WAVEGUIDE DETAILS

Two representative partially-suspended waveguides are shown in Fig. S3a and S3b, respectively. The waveguide entry and exit facets are 40 μm wide and 5 μm thick. By doing a Fourier transform of the end facet geometry, we get an angular distribution of light field from the waveguide shown in Fig. S3c. Using the half acceptance angle limit of 26.6° set by the f-1 1-inch focal length OAP, we calculate an amplitude input coupling efficiency of 0.49. For signal collection from the exit facet of the waveguide, we used a 4-inch focal length 1-inch diameter OAP. This gives the half acceptance angle limit about 7° and an amplitude collection efficiency of 0.1. By taking into account the facet reflection losses of 0.36, the power input coupling efficiency is estimated to be 10%, and the estimated collection efficiency is 0.5%.

The waveguide loss was calculated by using transmission spectroscopy on the waveguide. Assuming it acts as a Fabry-Perot cavity, the losses can be found using $\alpha = -\frac{1}{L}\ln\left(\frac{1}{R}\frac{\sqrt{\zeta}-1}{\sqrt{\zeta}+1}\right)$, where $\zeta = \frac{I_{max}}{I_{min}}$, R is the facet reflectivity of 0.36, and L is the total waveguide length of 0.4 cm.[1] At around 1288.5 cm^{-1}, the waveguide loss is calculated to be 6 cm^{-1}. At 1287.5 cm^{-1}, it is calculated to be 9 cm^{-1}.

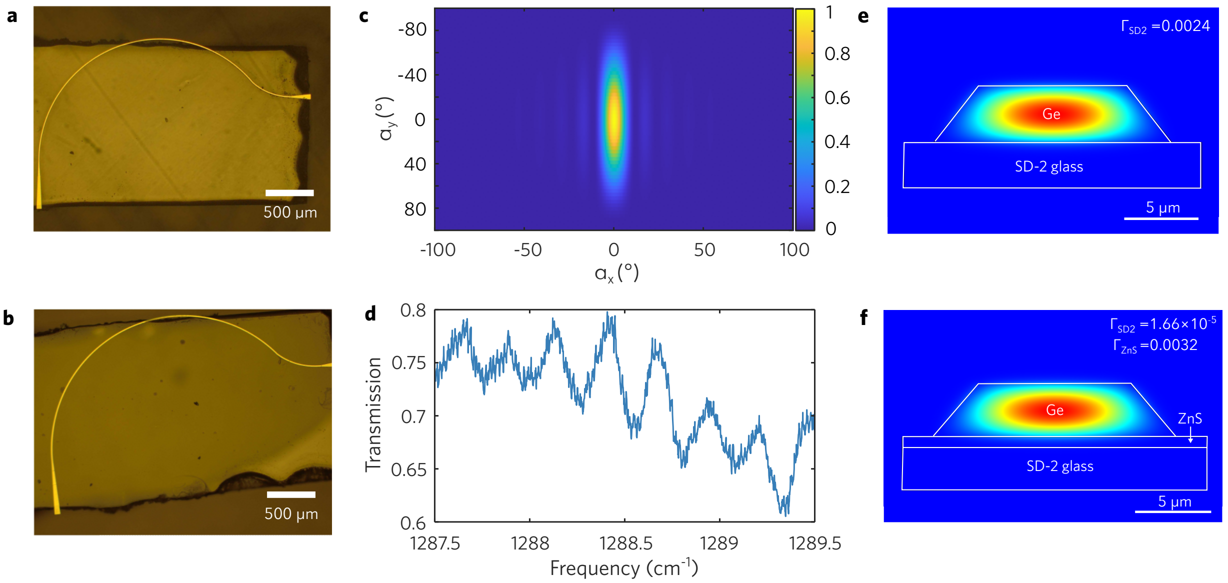

Figure S3. a, **b**. Optical images of two waveguides. **c**. Fourier transform of the waveguide entry facet, from which the coupling is calculated. **d**. Transmission of a waveguide relative to free space from 1287.5 to 1289.5 cm^{-1}, from which the net losses are computed. **e**. Schematic of the waveguides as they are currently fabricated. **f**. Schematic of waveguides fabricated on buffer film, which could in principle drastically lower their losses.

While this work utilized anodic bonding glass (Hoya SD-2) for its ease of integration with germanium, it is a very high-loss material in the LWIR (with losses estimated to be in excess of 3,500 cm^{-1}). However, as it only provides mechanical support, significant improvement in the losses could be obtained with moderate-loss buffers. While very few materials have losses as low as pure germanium, there are a number of materials, such as ZnS, ZnSe, and MgF_2, that have moderate losses and low refractive indices. Due to the high index of germanium, using them as a buffer layer could provide a substantial reduction in loss.

For example, Figure S3 shows the simulated mode profile of germanium directly on glass (Fig. S3e) and with a 1 μm of ZnS (n=2.2), which can be deposited on glass with high quality (Fig. S3f)[2]. The overlap of the mode with the glass in the first case is just 0.2%, but it is responsible for the observed losses of 6-9 cm^{-1}. With ZnS, however, the overlap with the glass would be pushed down to just 1.66×10^{-5}, pushing down excess losses to 0.04-0.06 cm^{-1}. The ZnS itself only has an overlap of 0.3%, so its material loss of 0.2 cm^{-1} would add negligible excess loss. However, even similar-index material with losses of 20 cm^{-1} would add only 0.06 cm^{-1} of additional modal loss.

III WATER VAPOR ABSORPTION LINES

We used the simulated water vapor absorption lines by using isotopologues 161, 181, 171, and 162 at 296 K and 1 atm pressure to calibrate the acquired spectra. The simulated three water vapor absorption lines from 1285 to 1290 cm^{-1} are located at 1287.40006 cm^{-1}, 1288.24992 cm^{-1}, and 1288.88653 cm^{-1}, respectively, shown in Fig. S4.

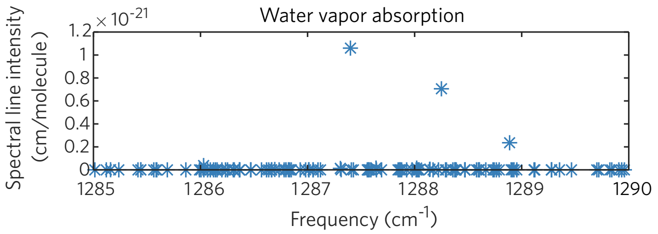

Figure S4. Vapor absorption lines simulated using HITRAN[3].

IV Modifying the waveguide-resonator coupling

From coupled-mode theory, when the waveguide is placed near the edge of the resonator, the coupling strength of the fundamental mode is significantly larger than the higher-order modes due to increased modal overlap. Transmission dips with less coupling strength correspond to higher-order mode families, and their overlap becomes more significant as the resonator is moved closer. In Fig. S5, the transmission dips of the fundamental mode show less than 10% transmission, indicating that the waveguide is close to critically-coupled for the fundamental mode. The transmission dips of the higher-order modes exhibit 20% to 30% transmission, as their coupling is reduced.

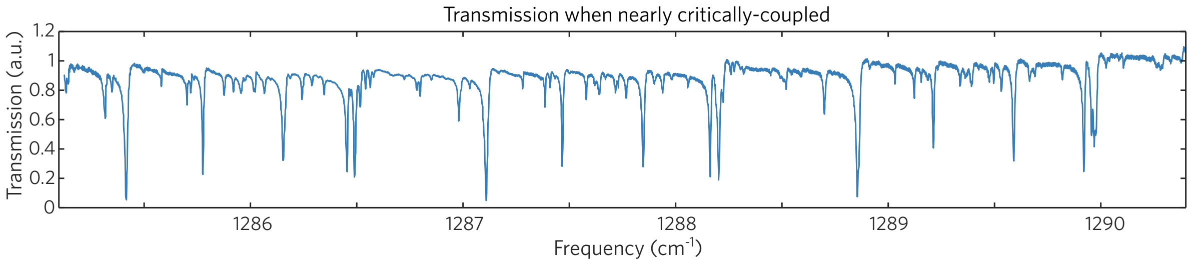

Figure S5. Representative normalized transmission spectrum when the waveguide is nearly critically-coupled to the resonator.

To evaluate the Q factors from the WGM microresonator systematically, we performed statistical analysis of Q values in the under-coupled condition with variable coupling strength. As is shown in Fig. S6a, the coupling strength is enhanced by tuning down the piezoelectric actuator voltage, on which the microresonator was mounted. Fits were performed to each of the measurements in the shaded area. The histogram is shown in Fig. S6b, with an average Q factor of 2.2×10^5.

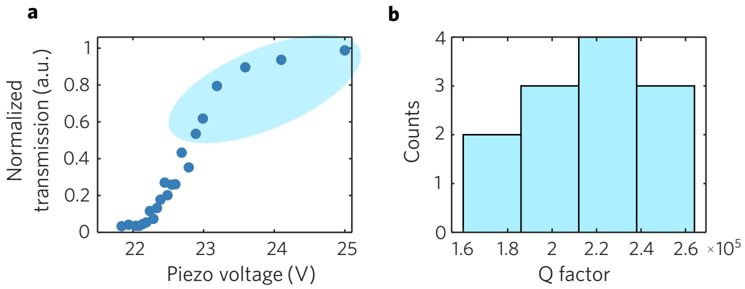

Figure S6. a. Points (measurements) counted in the shaded area for the statistics from the transmission (TE polarization) vs. piezoelectric actuator voltage chart. **b**. The histogram of Q values is taken from the points (measurements) in the shaded area from a.

V Optical losses of Si and Ge in the LWIR

To truly see the benefits of high-quality Ge for LWIR photonics, especially the non-epitaxially-grown platform and suspended WGM microresonator deveoped in this study, we have plotted the optical losses versus frequency from 550 to 1600 cm^{-1} in Fig. S7 using the data from Refs.[4–7]. Si has significant losses below 1500 cm^{-1} due to three-phonon absorption, limiting its

wide application for LWIR integrated photonics. In comparison, Ge has optical losses below 1 dB/cm down to 1000 cm^{-1}. Our WGM microresonators are indicated by the circle in Fig. S7 and have losses approximately four times lower than the theoretical losses of suspended silicon.

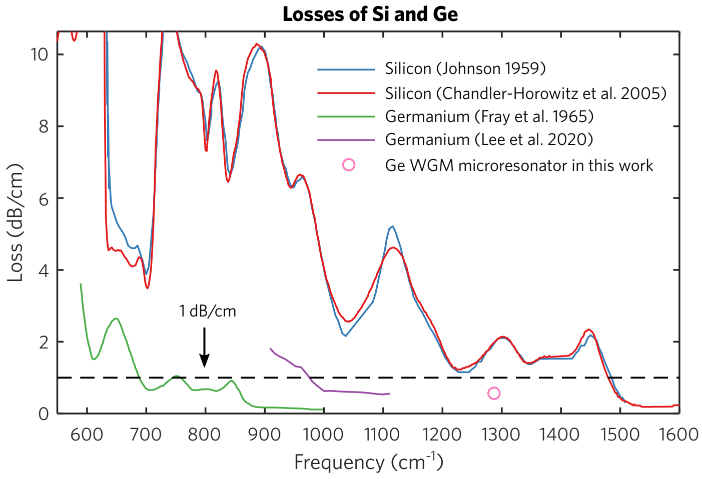

Figure S7. Optical losses of Si[4,6] and Ge[5,7] at LWIR.

VI PROTECTIVE DICING

The key for a damage-free dicing process comes firstly with a thick photoresist protective layer. As is seen by the process schematic in Fig. S8, we put a thick protective layer by directly dropping S1813 photoresist on the substrate without spinning. After baking at 90 °C for 6 hrs, the photoresist provides rigid protection against debris-induced damage to the microresonator. Then, the chip was diced about 50 μm away from the microresonator. If the dicing was too close, it might damage the microresonator. After the dicing process, the chip was directly put into the 49% HF etchant. Since the microresonator was protected by the thick photoresist, only the glass part of the diced edge was etched inwards towards the resonator. After 10 min in HF, the bottom glass at the edge of the microresonator close to the dicing facet was etched away, leaving the edge of the microresonator suspended over the chip edge.

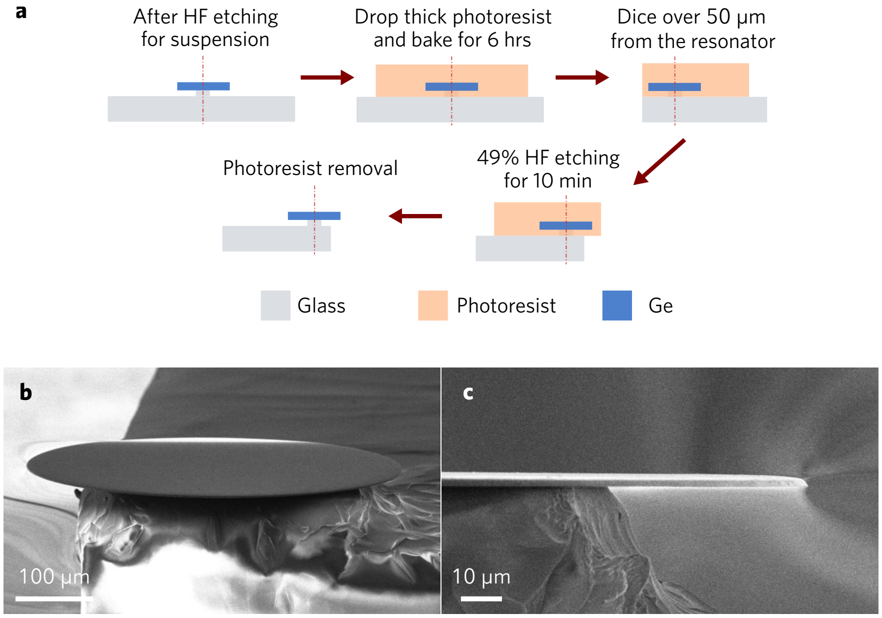

Figure S8. a. Side-view schematic of the protective dicing process. **b**. Bird's-eye view of an SEM of a microresonator suspended at the corner of a glass chip after dicing twice. **c**. Cross-sectional view of the WGM microresonator edge.

References

1. Gallacher, K. *et al.* Low loss Ge-on-Si waveguides operating in the 8–14 μm atmospheric transmission window. *Opt. Express* **26**, 25667–25675 (2018).

2. Benyahia, K., Benhaya, A. & Aida, M. S. ZnS thin films deposition by thermal evaporation for photovoltaic applications. *J. Semicond.* **36**, 103001 (2015).

3. Gordon, I. E. *et al.* The Hitran2020 molecular spectroscopic database. *J. Quant. Spectrosc. Radiat. Transf.* **277**, 107949 (2021).

4. Chandler-Horowitz, D. & Amirtharaj, P. M. High-accuracy, midinfrared (450 $cm^{-1} \leq \omega \leq 4000\ cm^{-1}$ refractive index values of silicon. *J. Appl. Phys.* **97**, 123526 (2005).

5. Fray, S., Johnson, F., Quarrington, J. & Williams, N. Lattice bands in germanium. *Proc. Phys. Soc.* **85**, 153 (1965).

6. Johnson, F. Lattice absorption bands in silicon. *Proc. Phys. Soc.* **73**, 265 (1959).

7. Lee, Y.-J., Das, A., Mah, M. L. & Talghader, J. J. Long-wave infrared absorption measurement of undoped germanium using photothermal common-path interferometry. *Appl. Opt.* **59**, 3494–3497 (2020).
